# Psychological and behavioural factors associated with sexual risk behaviour among Slovak students

**DOI:** 10.1186/1471-2458-9-15

**Published:** 2009-01-13

**Authors:** Ondrej Kalina, Andrea M Geckova, Pavol Jarcuska, Olga Orosova, Jitse P van Dijk, Sijmen A Reijneveld

**Affiliations:** 1 Kosice Institute for Society and Health. Department of Educational Psychology and Health Psychology, Faculty of Arts, P.J. Safarik University, Moyzesova 50, Kosice, 04059, Slovakia; 2 Department of Infectious Diseases, Faculty of Medicine, P.J. Safarik University, Tr. SNP 1, Kosice, 04066, Slovakia; 3 Department of Health Sciences, University Medical Center Groningen, University of Groningen, PO Box 30,001, 9700 RB Groningen, the Netherlands

## Abstract

**Background:**

Knowledge about the prevalence of sexual risk behaviour (SRB) in adolescence is needed to prevent unwanted health consequences. Studies on SRB among adolescents in Central Europe are rare and mostly rely on a single indicator for SRB. This study aims to assess the association of behavioural and psychological factors with three types of SRB in adolescents in Central Europe.

**Methods:**

We obtained data on behavioural factors (having been drunk during previous month, smoking during previous week, early sexual initiation), psychological factors (self-esteem, well-being, extroversion, neuroticism, religiousness), and SRB (intercourse under risky conditions, multiple sexual partners, and inconsistent condom use) in 832 Slovak university students (response 94.3%).

**Results:**

Among those with sexual experience (62%), inconsistent condom use was the most prevalent risk behaviour (81% in females, 72% in males). With the exception of having been drunk in males, no factor was associated with inconsistent condom use. Regarding the other types of SRB, early sexual initiation was most strongly associated. In addition, other, mostly behavioural, factors were associated, in particular having been drunk.

**Conclusion:**

Results suggest that behavioural factors are more closely related to SRB than psychological factors. Associations differ by type of SRB and gender but offer few clues to target risk groups for inconsistent condom use. Results show a high need for health-promotion programmes in early adolescence that target SRB in conjunction with other health risk behaviours such as alcohol abuse.

## Background

Studies on sexual behaviour from Central and Eastern Europe (CEE) are scarce. The lack of information on sexual behaviour is most salient regarding late adolescence and young adulthood, when young people start to live without direct parental supervision. The best recent information available is based on the Health Behaviour in School-aged Children (HBSC) studies 2001/2002 and 2005/2006 [1]. In both studies (Slovakia is not included in the first one) adolescents from Central and Eastern Europe reported that they were less experienced with sexual intercourse, used contraception pills or condoms during their most recent sexual intercourse to a lesser degree and initiated sexual intercourse later in life than their peers from most Western countries. However, patterns in the sexual behaviour of adolescents and young adults in Central and Eastern Europe seem to be changing. A decrease in the age at which they become sexually active is evident, particularly among females, leading to a narrowing of the gap between boys and girls regarding the time of sexual initiation.

In the context of sexually-transmitted infections (STI), many studies show the inconsistent use of prophylactic methods (e.g. condoms) to be the main risk factor [2-4]. However, early sexual intercourse, having multiple sexual partners and the association with substance use should also be considered as significant risk factors in this age group. Although each of these factors can be considered as an aspect of risk-taking, none by itself is valid as an operationalisation of risk behaviour [5,6]; yet all have become important topics in health promotion.

The association of sexual risk behaviour (SRB) with a number of other risk behaviours, including substance use, is evident. Use of marijuana, cocaine or other illicit drugs by adolescents has been shown to be associated with increased rates of sexual intercourse in general, having multiple sexual partners and lower rates of condom use, particularly for users of illicit stimulant drugs [7]. Binge-drinking teens are approximately three times less likely to use condoms, and recent marijuana users are almost two times less likely to use condoms [8]. The increasing prevalence of alcohol, drugs and tobacco use among Slovak adolescents as reported in the ESPAD (European School Survey Project on Alcohol and Other Drugs) reports of 1995, 1999, 2003, 2007, [9-12] and the lack of scientific studies in this field in CEE countries led us to explore the association among this behaviour and sexual risk behaviour.

Early sexual initiation is related to multiple aspects of SRB, including inconsistent condom use, early pregnancy and a greater number of sexual partners [13,14]. Moreover, it is also a predictor of future gynaecological problems. Girls who reported having sexual intercourse before age 16 had significantly more symptoms such as vaginal discharge and pruritus and signs such as abnormal discharge, erythema of the vaginal mucosa and lower genital tract infections than girls who first experienced sexual intercourse after age 19 [15]. It was found that 81% of sexually experienced youth aged 12–14 wished they had waited longer to have sex, compared with 55% of sexually experienced 15 to 19-year-olds [16]. Because of this we expect higher levels of SRB among those who report early sexual initiation.

SRB and other health-endangering behaviours may be considered the result of a number of determinants, which range from causal factors very close to the behaviour like attitudes and perceived social norms [17,18] to more distant causal factors like personality,[14,19,20] or even socioeconomic position [21]. As such, they may have shared causes with problem behaviour, as suggested by Jessor [17,18]. Thus, we might expect, for example, a co-occurrence with other problem behaviours,[7,22] or with psychological factors.

A study by Reitman [20] which explored the role of self-efficacy and self-esteem found that adolescents who believe they could take "effective precautionary action to avoid HIV" had fewer sexual partners and reported more condom use than peers who had lower self-efficacy scores [20]. Low self-esteem has also been associated with inconsistent use of contraceptives among adolescent girls [14]. Various indicators of psychosocial distress, which frequently occur along with low self-esteem, have been found to be associated with more frequent sexual activity [23-26]. Furthermore, several studies included religiousness among the factors associated with SRB, although the results from these studies are not consistent.

Summing up, the aim of our study was to explore the association of behavioural (drinking, smoking, early sexual intercourse) as well as psychological factors (self-esteem, psychological well-being, extroversion, neuroticism, religiousness) with three aspects of SRB: (1) sexual intercourse under risky conditions, (2) multiple sexual partners and (3) inconsistent condom use among late adolescents.

## Methods

### Sample

Data were collected in April and November 2004. The sample consisted of 882 first-year students at two universities located in Kosice (230,000 inhabitants) P.J. Safarik University (7,000 students) and the Technical University (12,000 students) who during a compulsory lecture completed a questionnaire concerning health behaviour under the guidance of field workers. Students were recruited from a list of randomly selected study groups provided by the faculties concerned and their participation was voluntary. All procedures concerning data collection were explained to respondents before data collection. The Ethics Committee of the Medical Faculty of the P.J. Safarik University approved this study. Of the 882 students included, 7 left the room before the beginning and 43 were excluded afterwards because they left major parts of the questionnaire incomplete (altogether 50). A total of 832 responded (94.3%), 355 male and 477 female, aged 19–28 years with 90% of the students aged 19–23 years (mean 20.5; SD 1.4). Out of these, 45.1% studied at the science faculty, 34.8% at the technical faculty and 20.1% at the medical faculty. More than half of the respondents had completed grammar school, and the majority of the students lived in student halls of residence or with their parents.

### Measures

Regarding *SRB*, respondents were asked (1) if they had had sex (penetration of vagina by the penis) after a short relationship or under the influence of drugs or alcohol (yes/no to any of the three conditions); (2) how many sexual partners they had had in their life (3 and less/4 or more); (3) how often they used condoms (always/almost always, occasionally, never).

Behavioural factors concerned binge drinking, smoking and early experience of sexual intercourse. Respondents were asked (1) how many times they had been drunk during the previous month (never/1 or more); (2) how many cigarettes they had smoked during the previous week (none/one or more) and (3) at what age they had had sexual intercourse for the first time. Those who had been drunk at least once during last month, smoked at least one cigarette per week and had sex before the age of 16 were indicated as behaving riskily. Categorisations regarding number of sexual partners, drunkenness and smoking were similar to ones that have been used previously [15,27-32].

Regarding *psychological factors*, self-esteem was assessed using the Rosenberg self-esteem scale [33]. The scale consists of 10 items (5 positive and 5 negative). Each item has a four-point scale ranging from "strongly agree" to "strongly disagree". For each question, the respondents choose the statement that most closely applies to them. The sum score for self-esteem varies from 10 to 40, a higher score indicating higher self-esteem. This variable was trichotomised into high (30 to 40), middle (20 to 29) and low (10 to 19).

Psychological well-being was measured with the shortened 12-item version of the General Health Questionnaire (GHQ12) [34]. The separate items focus on various aspects of respondents' psychological dispositions, for example problems with sleep, strain, happiness or stress. The questions compare how the respondents' present state differs from their usual state. The GHQ12 was scored using a four-point Likert scale (0, 1, 2, 3) with sum scores ranging from 0–36. A higher sum score means lower psychological well-being. The values were trichotomised into high (0 to 11), middle (12 to 23) and low (24 to 36) psychological well-being.

Extroversion and neuroticism were measured with an abbreviated form of the revised Eysenck Personal Questionnaire [35]. Extroversion was measured with a 6-item scale (yes/no) as was neuroticism. The sum score for extroversion/neuroticism varies from 6 to 12, with a higher score indicating lower levels of extroversion/neuroticism. Both variables were categorized into three levels: high (6 to 7), middle (8 to 10) and low (11 to 12) extroversion/neuroticism.

One item of the Questionnaire for Instrumental and Terminal Values [36] was used to measure religiousness. Respondents were asked to evaluate how important salvation (feeling of redemption, eternal life) is for them (1-extremely important, 2-strongly important, 3-important, 4-less important and 5-unimportant). A higher score for this value indicates lower religiousness in the respondent. This variable was categorized into two levels: high (extremely important and strongly important) and low (important, less important and unimportant).

### Statistical analyses

We first examined the proportion of students that had had sexual intercourse. Next, for those who had had intercourse at least once (n = 455), we examined, using logistic regression, the association of each indicator of SRB with behavioural and psychological factors separately for males and females. We computed crude odds ratios for each type of SRB in relation to each factor. Subsequently, we determined the mutually adjusted associations of factors with SRB by forward selection procedures, starting with all factors that had a statistically significant crude odds ratio. We repeated these analyses with addition of age into the models, which yielded very similar results (not shown). All analyses were done with SPSS software, version 14.00.

## Results

Table 1 presents a descriptive view of the behavioural and psychological factors separately for males and females. Of the 832 respondents, 455 reported having had sexual intercourse. Out of these, 44% of males versus 33% of females said they had had sex under risky conditions; 27% of males versus 21% of females said they had had 4 or more sexual partners in their life; 72% of males versus 81% of females reported inconsistent condom use and 9% of males versus 5% of females had had sex before age 16.

**Table 1:** Sexual risk behaviour and other risk behaviour (n = 832)

	Male (n = 184)		Female (n = 271)	
	Number	%	Number	%
Sexual experience *				
Yes	184	61.3	271	63.3
**Having sex before age of 16**				
Yes	15	8.5	13	5.1
**Having sex under risky conditions**				
Yes	81	44.3	87	32.8
**Multiple sexual partners**				
Yes	45	26.5	54	21.4
**Inconsistent condom use**				
Yes	131	72.4	209	80.7
**Being drunk at least once during last month**				
Yes	88	48.1	79	29.3
**Smoking one cigarette at least once per week**				
Yes	80	44.2	101	38.3
**Self-esteem**				
High	51	28.7	66	25.3
Middle	100	56.2	134	51.3
Low	27	15.2	61	23.4
**Psychological well-being**				
High	51	27.9	56	20.9
Middle	102	55.7	132	49.3
Low	30	16.4	80	29.9
**Extroversion**				
Low	21	11.9	36	14.1
Middle	87	49.4	114	44.7
High	68	38.6	105	41.2
**Neuroticism**				
Low	60	32.8	47	17.9
Middle	90	49.2	131	49.8
High	33	18.0	85	32.3
**Religiousness**				
Strongly important	65	43.0	95	42.6
Unimportant	86	57.0	128	57.4

* from whole sample – male (n = 355), females (n = 477)

Tables 2 and 3 present the results of the logistic regression models analysing the associations of each indicator of SRB with the behavioural and psychological factors separately for males and females. Statistically significant (*p*<0.05) associations are summarized below.

**Table 2:** Determinants of SRB in males (n = 184): odds ratios (OR) and 95%-confidence intervals (CI)

	Sex in risky conditions				Multiple sexual partners				Inconsistent condom use			
	**n**	%	OR	95% CI	**n**	%	OR	95% CI	**n**	%	OR	95% CI
**Being drunk at least once during last month**												
No	32	33.7	1.00		17	19.3	1.00		61	64.9	1.00	
Yes	48	55.2	2.42**	1.33–4.41	28	34.6	2.21*	1.10–4.44	69	80.2	2.20*	1.11–4.33
**Smoke at least one cigarette per week**												
No	44	43.6	1.00		17	18.3	1.00		69	69.0	1.00	
Yes	36	45.6	1.09	0.60–1.96	27	36.5	2.57	1.27–5.21	60	76.9	1.50	0.76–2.94
**Having sex before age of 16**												
No	66	41.0	1.00		37	23.9	1.00		114	70.8	1.00	
Yes	12	80.0	5.76**	1.56–21.20	8	66.7	6.38**	1.82–22.39	13	86.7	2.68	0.58–12.34
**Self-esteem**												
High	19	37.3	1.00		16	33.3	1.00		33	66.0	1.00	
Middle	46	46.5	1.46	0.73–2.92	23	25.3	0.68	0.32–1.45	76	77.6	1.78	0.84–3.78
Low	12	44.4	1.35	0.52–3.48	5	19.2	0.48	0.15–1.50	18	66.7	1.03	0.38–2.78
**Well-being**												
High	20	40.0	1.00		14	32.6	1.00		33	66.0	1.00	
Middle	50	49.0	1.44	0.73–2.87	23	24.0	0.65	0.30–1.44	77	77.0	1.73	0.82–3.64
Low	10	33.3	0.75	0.29–1.93	8	26.7	0.75	0.27–2.11	20	66.7	1.03	0.40–2.69
**Extroversion**												
Low	7	33.3	1.00		3	14.3	1.00		14	70.0	1.00	
Middle	33	38.4	1.25	0.46–3.41	14	17.7	1.29	0.33–4.99	59	69.4	0.97	0.34–2.81
High	36	52.9	2.25	0.81–6.27	26	40.6	4.11*	1.10–15.37	50	73.5	1.19	0.40–3.57
**Neuroticism**												
Low	25	41.7	1.00		15	27.3	1.00		47	78.3	1.00	
Middle	43	48.3	1.31	0.68–2.53	21	25.0	0.89	0.41–1.92	58	66.7	0.55	0.26–1.18
High	13	39.4	0.91	0.38–2.17	9	29.0	1.09	0.41–2.90	25	75.8	0.86	0.32–2.36
**Religiousness**												
Strongly important	30	46.9	1.00		22	36.1	1.00		45	70.3	1.00	
Unimportant	42	48.8	1.08	0.57–2.07	17	21.8	0.49	0.23–1.05	63	75.0	1.21	0.59–2.49

* p. < .05. ** p. < .01 ***p. < .001

**Table 3:** Determinants of SRB in females (n = 271): odds ratios (OR) and 95%-confidence intervals (CI)

	Sex in risky conditions				Multiple sexual partners				Inconsistent condom use			
	
	**n**	%	OR	95% CI	**n**	%	OR	95% CI	**n**	%	OR	95% CI
**Being drunk at least once during last month**												
No	45	24.2	1.00		34	19.1	1.00		141	77.9	1.00	
Yes	41	52.6	3.47***	1.99–6.06	20	27.4	1.60	0.85–3.02	68	87.2	1.93	0.91–4.09
**Smoke at least one cigarette per week**												
No	33	20.9	1.00		21	14.0	1.00		118	76.1	1.00	
Yes	53	53.0	4.27***	2.47–7.40	33	34.7	3.27***	1.75–6.11	86	88.7	2.45*	1.18–5.08
**Having sex before age of 16**												
No	73	30.2	1.00		47	19.8	1.00		189	79.7	1.00	
Yes	11	84.6	12.73***	2.75–58.89	7	53.8	4.72**	1.51–14.69	12	92.3	3.05	0.39–24.02
**Self-esteem**												
High	21	31.8	1.00		14	21.9	1.00		51	79.7	1.00	
Middle	47	35.6	1.19	0.63–2.22	28	22.0	1.01	0.49–2.09	102	79.1	0.96	0.46–2.02
Low	16	27.6	0.82	0.38–1.77	11	21.2	0.96	0.39–2.34	50	87.7	1.82	0.67–4.94
**Well-being**												
High	19	33.9	1.00		15	27.3	1.00		42	75.0	1.00	
Middle	39	30.2	0.84	0.43–1.65	28	23.1	0.80	0.39–1.66	101	81.5	1.46	0.69–3.12
Low	29	37.7	1.18	0.57–2.42	11	15.1	0.47	0.20–1.13	64	84.2	1.78	0.75–4.22
**Extroversion**												
Low	6	17.1	1.00		2	5.9	1.00		26	76.5	1.00	
Middle	29	25.9	1.69	0.64–4.48	18	16.8	3.24	0.71–14.73	88	78.6	1.13	0.45–2.81
High	48	47.1	4.30**	1.64–11.23	33	34.7	8.52**	1.92–37.76	81	83.5	1.56	0.60–4.06
**Neuroticism**												
Low	12	25.5	1.00		9	20.0	1.00		35	77.8	1.00	
Middle	43	33.3	1.46	0.69–3.09	27	21.3	1.08	0.46–2.52	107	83.6	1.46	0.63–3.39
High	31	38.3	1.81	0.82–4.00	18	24.7	1.31	0.53–3.23	60	75.9	0.90	0.38–2.16
**Religiousness**												
Extremely important	25	26.3	1.00		10	11.0	1.00		75	79.8	1.00	
Unimportant	48	38.1	1.72	0.96–3.08	36	29.5	3.39**	1.58–7.28	98	79.0	0.96	0.49–1.85

* p. < .05. ** p. < .01 ***p. < .001

### Sex under risky conditions

Males and females who reported having been drunk at least once in the previous month or having had sex before the age of 16 were more likely to engage in sex under risky conditions. Moreover, girls who reported smoking and higher extroversion were more likely to engage in such types of sexual risk behaviour (see Tables 2, 3). Introducing these variables in a multiple logistic regression model with forward selection resulted in all of them being selected. The resulting mutually adjusted odds ratios are presented in Table 4.

**Table 4:** Factors associated with SRB after forward selection: odds ratios (OR) and 95%-confidence intervals (CI) ^1^

	Sex in risky conditions		Multiple sexual partners	
**Males**	OR	95% CI	OR	95% CI
**Being drunk at least once during last month**				
No	**1.00**	**	**1.00**	**
Yes	**2.48**	1.32–4.64	**2.25**	1.04–4.88
**Having sex before age of 16**				
No	**1.00**	**	**1.00**	**
Yes	**5.97**	1.58–22.53	**6.41**	1.73–23.80
**Extroversion**				
Low			**1.00**	*
Middle			**1.22**	0.29–5.10
High			**3.50**	0.87–14.13
				
**Females**				

**Being drunk at least once during last month**				
No	**1.00**	**		
Yes	**2.41**	1.30–4.46		
**Smoke at least one cigarette per week**				
No	**1.00**	***	**1.00**	*
Yes	**3.00**	1.66–5.44	**2.55**	1.20–5.40
**Having sex before age of 16**				
No	**1.00**	*	**1.00**	
Yes	**7.18**	1.46–35.38	**3.57**	0.91–14.02
**Extroversion**				
Low	**1.00**	*	**1.00**	**
Middle	**1.42**	0.48–4.17	**2.80**	0.56–14.16
High	**2.96**	1.03–8.48	**8.22**	1.69–40.03
**Religiousness**				
Extremely important			**1.00**	*
Unimportant			**2.82**	1.24–6.45

^1 ^ORs in bold indicates that overall a variable contributes to the logistic model at * p. < .05. ** p. < .01 ***p. < .001

### Multiple sexual partners

The risk of multiple sexual partners was associated with more psychological factors among females. Males reporting having been drunk at least once in the preceding month or reporting sexual experience before age 16 were more likely to have had more than 3 sexual partners in their life. Extroversion was associated with multiple sexual partners among males, with a rather high though not statistically significant OR for the 'high extroversion' category.

Females reporting smoking or reporting sexual experience before age 16 were more likely to have had more than 3 sexual partners. Females reporting high extroversion and religiousness as unimportant were more likely to have had multiple sexual partners in comparison to their peers reporting a low level of extroversion or a high level of religiousness. Introducing these variables into a multiple logistic regression model with forward selection resulted in all of them being significant, except for having sex before age of 16 in the female sample. The resulting mutually adjusted odds ratios are presented in Table 4.

### Inconsistent condom use

Only one behavioural factor in males (being drunk) and one in females (smoking) was associated with inconsistent condom use. Introducing this variable into a multiple logistic regression model with forward selection resulted in selection of only this variable.

## Discussion

This study on SRB in young adults in CEE shows that the occurrence of SRB in Slovak young adults varies from 21 to 81% of sexually experienced respondents (n = 455), depending on the indicator used. The most frequently reported SRB is inconsistent condom use, despite the fact that consistent condom use is one of the most efficient ways of protection. The occurrence of sexual intercourse under risky conditions is also very high, varying between 33–44% of the sexually experienced. From 5 to 9% of respondents reported having already had sex before age 16, and 27% of males and 21% of females reported having had multiple sexual partners.

From an international point of view, Slovak students have their sexual initiation exceptionally late, and the number of their sexual partners is low. On the other hand, similar to other studies, no gender differences were found in the prevalence of having sex. These findings fit with those from the HBSC study, where a lower proportion of sexually experienced individuals was reported among 14 and 15 years old adolescents in Central European countries (Hungary, Czech Republic, Croatia, Poland) compared with adolescents from Western or Northern European countries [1].

In line with several other studies [37-40], our study shows that alcohol use is one of the most consistent predictors of SRB. This finding supports the explanations that less self-control leads to risk behaviour and that certain people have a psychological predisposition to seek sensation and are thus more likely than others to engage in a variety of risk behaviours [41]. However, without contextual information about this event, we cannot clearly state that alcohol or drug use has a causal relation to SRB. It should be noted that our measurement of having sex under risky conditions covers lifetime history, which might be the reason for the high proportion, but on the other hand, lifetime history in this age group represents only about 4 years of life. Future analyses should go more deeply into the dimensions of a relationship like trust, intimacy, commitment and communication and their effect on behaviour. Our approach is important especially among the young population, where the age of the first contact with alcohol consumption is rapidly dropping [1].

The study results also revealed relevant differences between male and female sexual behaviour. Males report mostly behavioural factors as significant predictors for sex under risky conditions and multiple sexual partners. On the other hand, females add psychological factors. Their lower levels of extroversion and higher levels of religiousness are associated with fewer sexual partners, in accordance with other studies [42,43], where religiousness seems to be a protective factor against a high number of sexual partners among girls. On the border of statistical significance was the association between a high level of religiousness and a low probability of having sex under risky conditions. Thus, it is probably not only religiousness that plays an important role. Several studies have shown that girls who report a high importance of religiousness probably live in such psychosocial and cultural environments (personality, relationships, family, friends, and school) that opportunities for SRB are rather limited [44,45]. On the other hand religiousness had no significant association with sex under risky conditions or inconsistent condom use, which suggests that among those who have already had sexual intercourse, religiousness does not play a role. We can conclude, then, that despite the high importance of religiousness among students, their sexual behaviour reflects strategies more risky than safe.

Inconsistent condom use has been frequently reported as a major pattern of SRB. Only those who reported consistent condom use were defined as a safe group; all others were defined as a risk group. This strict criterion comes from the assumption that each intercourse without a condom is risky in terms of STI infection or unintended pregnancy. Of course, such a definition cannot be used in each age group, but among first year university students, 90% of whom are in the 19–23 age range and where it is difficult to expect stable and long lasting sexual partnerships or unprotected intercourse with the aim of becoming pregnant, this methodical criterion should fit. Despite the high rates of inconsistent condom use in our sample (72.4% of males and 80.7% of females), we only found an association with drinking in males and smoking in females. These results are in accordance with previous research [8,46,47], which showed alcohol consumption to be negatively associated with condom use. Nevertheless, recent studies have recognized the importance of examining how sexual relationships themselves influence condom use. It is possible that alcohol only has an effect on condom use at specific phases in a relationship [48].

Studies which explored the role of alcohol use on condom use [49-51] and studies which assessed the length of a relationship [52] or the type of relationship [53] did not find any association between alcohol consumption and condom use. Moreover, these results do not support the persistent notion that alcohol causes people to engage in sexual risk that they would avoid when sober. Instead, people tend to follow their usual pattern of condom use, regardless of alcohol use [50]. Such inconsistent findings regarding condom use and drinking suggest the possible effect of a third factor which affects both variances, and that any relationship between condom use and drinking is disputable. Mental health problems, developmental factors, disposition to risk taking and sensation seeking, familial influences and general tolerance for deviance have all been reported as possible third variances in literature sources [54-57]. Consequently, an association between alcohol use and condom use could be attributable to these factors and not to any relationship between alcohol and condom use [48]. Nevertheless, our results suggest that behavioural factors are more closely related to SRB than psychological ones.

### Methodological considerations

This study has several strengths and limitations. Due to possible methodological problems, studies of sexual behaviour in CEE countries are rare. We obtained a very high response rate (94%), however, by using the setting of lectures, so selection bias is very unlikely to occur. We cannot exclude information bias, however, though we did use specific measures to guarantee confidentiality. These measures have been shown to yield valid outcomes.

Regarding multiple sexual partners among males, the combination of 95%-confidence intervals of both the 'middle' and the 'high' categories of extroversion comprising '1' (i.e. are not statistically significant different from the reference category) but at the same time extroversion contributing to the model with statistical significance seems odd. It can be explained by the fact that the associations of the middle and the high categories with the outcome differ quite a lot too. The latter has been taken into account regarding the overall p-value, but not regarding the comparison of these separate categories with the same reference category (i.e. 'low') and the resulting 95%-confidencence intervals. This holds for any logistic regression in which dummy coding is used for separate categories (like we did).

### Implications

Our findings support the hypothesis that risk behaviours tend to cumulate, e.g. sexual risk behaviour may coincide with binge drinking and smoking. However, one of our indicators for SRB (having sex under risky conditions, e.g. after a short relationship or under the influence of drug or alcohol consumption) may overlap with one of the explored independent variables (being drunk). To understand whether alcohol has an effect on adolescents' condom use, future research should consider whether adolescents are drinking at the time that the decision is made, because it may be that alcohol negates any skill learned while sober [48].

Additional research is needed to assess whether other factors so we may suppose that smoking or binge drinking increase the contribute to consistent use of condoms, such as the level of health awareness, self-efficacy and anxiety related to health risk, or participation in SRB while accepting the risk involved in such behaviour. However, we did not find any associations between self-esteem and consistent or inconsistent condom use, which contrasts with the findings of several studies [58,59]. Particularly in this case, our findings should be interpreted with this special aspect in mind. However, we confirmed the associations of drinking and smoking on SRB, probability of SRB, or, in other words, that they are risk indicators with regard to SRB in any case.

## Conclusion

The overall findings of our study suggest that the specification of SRB into three indicators contributes to a better understanding and description of SRB. All three indicators provide a specific and different view on adolescent sexual behaviour. Consequently, several significant differences were found between the indicators, which suggest an important variance in SRB and allows several recommendations to be formulated. Systematic prevention should be focused on the high incidence of sexual risk behaviour among young people, which indicates the need for health promotion programmes not only on smoking, alcohol and drugs, for example, but that sexual risk behaviour should also be integrated into prevention programmes. Due to the accumulation of risk behaviour among young people, focusing on prevention in a related set of unhealthy behaviours instead of a single type of unhealthy behaviour will be very important, particularly in early adolescence. Moreover, results show a high need for health promotion programmes in early adolescence that target SRB in conjunction with other health-related risk behaviours such as alcohol abuse.

## Competing interests

The authors declare that they have no competing interests.

## Authors' contributions

OK carried out the data collection, did the analysis, interpreted the data, and drafted the manuscript. AMG participated in the design and the coordination of the study and helped to draft the manuscript. PJ participated in the design of the study and questionnaire and helped to draft the manuscript. OO participated in the data collection, in coordination and helped to draft the manuscript. JPvD participated in the design of the study and helped to draft the manuscript. SAR commented on the design of the study, contributed to the statistical analysis and drafted the final version of the manuscript. All authors read and approved the final manuscript.

## Pre-publication history

The pre-publication history for this paper can be accessed here:


